# An Eye on the Future of COVID-19: Prediction of Likely Positive Cases and Fatality in India over a 30-Day Horizon Using the Prophet Model

**DOI:** 10.1017/dmp.2020.444

**Published:** 2020-11-18

**Authors:** Vatsal Tulshyan, Dolly Sharma, Mamta Mittal

**Affiliations:** 1 Department of Computer Science and Engineering, Amity School of Engineering and Technology, Amity University, Noida, India; 2 Department of Computer Science and Engineering, G. B. Pant Government Engineering College, Okhla, New Delhi, India

**Keywords:** COVID-19, health, prediction, Prophet model

## Abstract

**Objective:**

The coronavirus disease (COVID-19) pandemic was initiated in Wuhan Province of mainland China in December 2019 and has spread over the world. This study analyzes the effects of COVID-19 based on likely positive cases and fatality in India during and after the lockdown period from March 24, 2020, to May 24, 2020.

**Methods:**

Python has been used as the main programming language for data analysis and forecasting using the Prophet model, a time series analysis model. The data set has been preprocessed by grouping together the days for total numbers of cases and deaths on few selected dates and removing missing values present in some states.

**Results:**

The Prophet model performs better in terms of precision on the real data. Prediction depicts that, during the lockdown, the total cases were rising but in a controlled manner with an accuracy of 87%. After the relaxation of lockdown rules, the predictions have shown an obstreperous situation with an accuracy of 60%.

**Conclusion:**

The resilience could have been better if the lockdown with strict norms was continued without much relaxation. The situation after lockdown has been found to be uncertain as observed by the experimental study conducted in this work.

## Introduction

Coronavirus disease (COVID-19) is considered to be caused by a zoonotic virus because 2 earlier zoonotic viruses, severe acute respiratory syndrome (SARS) and Middle East respiratory syndrome (MERS), had traces from the bat. The reason behind COVID-19 was traced to the bat because the other viruses had a sequence similar to the new coronavirus and all of them were found similar to the one sampled from bats.^1–2^

This virus is said to belong to the crown family of viruses since it has crown-like projections that affect people with less immunity or having other diseases. People with this virus have said to exhibit common symptoms like dry cough, high fever, and breathlessness. This virus has an incubation period of 14 days and can be spread through respiratory droplets and close contact. Even during the latency period of the infection, it can also be contagious. The contagion can be controlled by taking mitigation measures at the right time.^3^

The new coronavirus has been declared a global pandemic and emergency by the World Health Organization on March 11, 2020.^4,5^ The said virus first spread across foreign countries, but, as foreigners and non-resident Indians started visiting India, cases in India also increased subsequently. The cases in India have been rising since March 8. As a preventive measure, a nationwide lockdown was imposed after March 24, 2020. It was important to take this measure, but it affected the economy’s developing country like India.^6^

The growth rate can be defined as the rate of change of a specific variable within a specific period. The growth rate of COVID-19 is 5.8 ~ 6 (average).^7^ The growth rate shows that a lockdown has been able to keep control over the current situation. It is consistent to date as of May 24. Being cautious about the situations and learning from other countries have helped India to control the contagion. India has not been battered like other countries in the world who have faced the worst disaster in terms of deaths. Given the health conditions and infrastructure, the country’s citizens are following the norms of the lockdown and the government has made arrangements of 1.8 million beds across the nation for the treatment of COVID-19 patients. The estimates of the case fatality rate or mortality rate are highly dependent on country-specific demographic criteria and various disease characteristics.^8^ On March 13, the first death in India was reported. Initially, the mortality rate was around 3% and later a decline was observed, resulting in the mortality rate of 2%. On May 24, 4021 deaths were reported with a total of 200 582 COVID-19-positive cases. The mortality rate of COVID-19 is ~2–3%, as compared with the mortality rate of ~14–15% of SARS.^9^

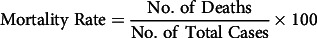



A few time series models have been used by researchers^9–13^ to forecast the total cases and deaths for a 30-day window. Some of them are the long short-term memory (LSTM) model,^10^ autoregressive integrated moving average (ARIMA) model,11 and the Susceptible-Infected-Recovered (SIR) model.^13^ These previous studies on the novel coronavirus have depicted the sudden uprise that would be expected in the coming months and have not considered relevant factors like lockdown, and unlocking and relaxation of the lockdown in multiple shifts. The factors like lockdown can have a prevailing effect on the forecast being made where people are expected to self-quarantine themselves.

The selection of the Prophet model^14^ is done for this research because it involves a lot of factors like holidays, seasonality, type of growth, and changepoints that can help in predicting efficiently, as analysts can have some assumptions made on the prediction. Thus, this model has provided a unique and exclusive approach toward time series forecasting using exponential smoothing. Also, it tends to perform better than others in terms of accuracy. India has been considered for this case study because the country has an average population density of 325 per square kilometers.^15^ The analysis of lockdown and unlocking in multiple shifts done over this country can help optimize solutions in the coming years if any such pandemic arises. Depending on how well we analyze the current situations in the affected countries, we will gain insights on how we can deal with such pandemics in the future. Hence, we arrive at the hypothesis regarding how much of an effect does lockdown and unlock have on the prediction of the total cases and deaths in the coming time.

## Methodology

This research is based on the Prophet model, which is generally used for business forecasting. As mentioned, this model includes parameters like holidays, trend, and seasonality, which would help mold the prediction results. The Prophet model is usually not used in the scope of predicting a pandemic, but we have lined the parameters to analyze predictions based on the Prophet model, such as using lockdown as holidays. This research is conducted in 2 phases. The first phase is conducted during the lockdown and the second phase, after the lockdown. During the lockdown phase, strict rules were imposed by the government and no relaxation was there. This phase enlightens the fact of how controlled the situation can be, keeping the complete lockdown rules in sight. This was one of the primary periods of concern for this research. The next circumstance, which needs to be understood, is the amount of change in the number of positive cases of COVID-19 that comes into account when laws are relaxed for the lockdown and people start returning to the new normal. This period is termed as *period after the lockdown*. Since the number of deaths is highly dependent on the total number of cases, it’s why the prediction made on deaths is done taking the whole period of lockdown into consideration.

COVID-19 miserably affected everyone’s lives, so there is a need for a prediction model that can forecast the situation and alert us to be better prepared for the future. This research has been performed using the data science website, www.kaggle.com (accessed May 24, 2020), using the data set named “COVID-19 in India.” The data set gets updated daily for cases for all states and union territories since January 30, 2020. For this research, the interval from March 24 to May 24 has been considered. As of May 24, 2020, the dimensions of the data are 2306 rows and 7 columns. The data set has been pre-processed by removing the missing values where no case for a state was recorded and total cases have been added as shown in Table 1. For the prediction purpose, a pivot table was obtained from the data set where all the total cases and deaths were summed cumulatively according to the dates. Python is used as the programming language.

**Table 1.** Data set of COVID-19 cases in India as of May 24, 2020

S. No.	Date	State/Union Territory	Cured	Deaths	Confirmed	Total Cases
1	03-24-20	Andhra Pradesh	0	0	8	8
2	03-24-20	Bihar	0	1	3	4
3	03-24-20	Chhattisgarh	0	0	1	1
⁞	⁞	⁞	⁞	⁞	⁞	⁞
2302	05-24-20	Tamil Nadu	7491	103	15 512	23 106
2303	05-24-20	Telangana	1065	49	1813	2927
2304	05-24-20	Tripura	153	0	189	342
2305	05-24-20	Uttarakhand	56	2	244	302
2306	05-24-20	Uttar Pradesh	3406	155	6017	9578

**Figure 1.**
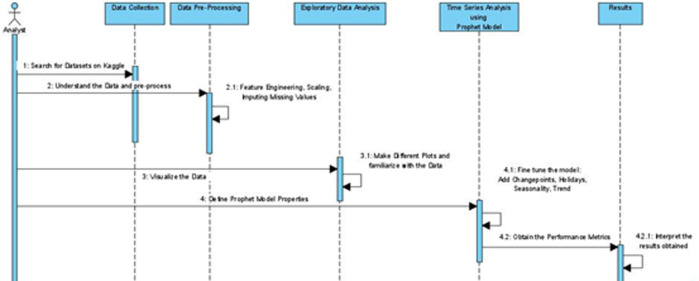
Sequence diagram for methodology.

Figure 1 demonstrates the procedure adopted for conducting this research.

Commonly used approaches for predicting the number of cases in epidemics are either time series analysis or the SIR model, where *S* stands for the number of people who are still at risk that the disease might infect them. The number of infected people at time *t* is given by *I(t)*. We assume that *I(0) > 0* because, otherwise, the epidemic cannot start. *R(t)* symbolizes the number of removed people at time *t*. In other words, the number of deaths that have been seen at time *t*.^14^ There are some limitations to this model. This model assumes that every person is moving and has an equal chance of getting in contact with every other person among the population, irrespective of the space or distance between them. It is assumed that the transmission rate remains constant throughout the pandemic. Moreover, this model does not cater to those infected who have been diagnosed or are in quarantine. It treats the same as those who have not been quarantined. Therefore, both are considered to have the same transmission rate. A time series is the sequence of one or more values per time step. This analysis can take the trend, seasonality, and other factors into account for forecasting purposes. There are some other methods that can be used for a time series analysis like the Prophet, LSTM, and ARIMA models.

In this research, the Prophet model has been used for the analysis and prediction as it has some merits over other models such as the ARIMA model; in the Prophet model, the interpolation of missing values is not required. Measurements need not be regularly spaced. Analysts can also add seasonality and holidays to improve the model performance and perhaps obtain a better prediction and forecasting. As in the case of COVID-19, the lockdown was imposed, which can be considered as an influencing factor toward the prediction. Analysts tend to have experience with regression and therefore can easily extend the model to include new components. Regression refers to a set of statistical processes for the estimation of the relationship between the target variable and 1 or more features. The Prophet model has interpretable parameters, which can be changed by the analyst to provide some assumptions for the prediction. The analyst can make assumptions on different trends. While using the ARIMA model, trend and seasonality need to be removed and later added while making the forecasts. There are some reasons in which Prophet performs better than the LSTM model. LSTM requires more data as compared with Prophet for a time series analysis in order to compute more accurate results. The reason behind LSTM requiring more data is that it needs to understand the non-linearity of the data provided. The Prophet model can add the seasonality and holiday parameters, which produce assumptions on the predictions. The Prophet model is easy to implement as compared with LSTM networks. LSTM networks do not learn the trend or seasonality of the data. The time complexity of the neural network that consists of LSTM is (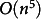), which is quite high as compared with the complexity of the Prophet model that is 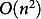.

The Prophet model was developed by Facebook in 2017.^14^ The forecasting has been done for the next 30 days starting from May 25, 2020. This model is based on the regression technique (Equation 1). This uses only time as a regressor, but several linear and non-linear functions of time can be used as components.(1)
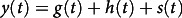



Here, 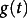 is the trend function that models non-periodic changes in the value of the time series, 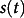 represents periodic changes (eg, weekly and yearly seasonality), and 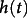 represents the effects of holidays that occur on potentially irregular schedules over 1 or more days.

Trend 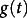 can be classified into 2 types: saturation and piecewise linear models.

Equation 2 represents the non-linear and saturation growth trend. 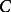 is the carrying capacity, 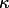 is the growth rate, and 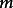 is an offset parameter. Carrying capacity is some maximum achievable point like the total market size, total population, and so on. An offset can be described as the location of a data point with respect to another location of a data point. The carrying capacity in this equation is replaced by a time-varying capacity C(t) because the capacity of any application is usually not constant, and it could change depending upon the time. The growth rate is also not constant. (2)
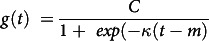



Piecewise Function (Equation 3) is the function that is broken down into parts where each part is based on change points provided.^14^
(3)
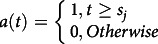

(4)
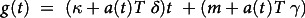



Equation 4 represents the Linear Trend with change points. Change points are defined as the points where the growth rate is allowed to change. In Equation 4, κ is the growth rate, δ is the rate of adjustment, 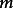 is the offset parameter, and 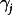 is set to 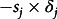 to make the function continuous; 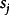 is a specific change point where 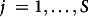. S is the total number of change points; 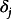 is the change in the rate that takes place at 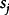.

Seasonality models are periodic functions of *t* (Equation 5). The Fourier series provide a flexible model of periodic effects. Let P be the regular period we expect the time series to have. For example, *P* = 365:25 for yearly data and *P* = 7 for weekly data, if we scale our time variable in days.(5)
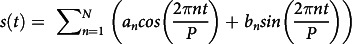



Holidays, *h(t)*, can be defined as time, *t*, during the holiday (Equations 6 and 7). Assign each holiday a parameter, 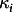, which is the corresponding change in the forecast. (6)
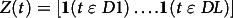
and taking(7)
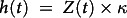



The parameters such as holidays, growth, and seasonality are responsible for the working of the Prophet model, which is evident from Equation 1. The Prophet procedure adopts an additive regressive model using 4 main components, that is, growth function, a yearly seasonal component modeled using Fourier series, a weekly seasonal component modeled using dummy variables, and analyst provided holidays list.

The horizon (*H*) is the number of days that the model is forecasting (Equation 8). This is typically 30, 60, 90, and so on, depending on the application that the user is trying to build. For any forecast that takes place to *H*, the forecast states will be produced with some error. Let 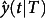 represent a forecast at time *t* made with historical information up to time *T*. Let 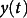 represent the actual value at time *t*. (8)
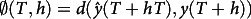



The reliability and validity of the prediction observed through the Prophet model is based on its performance metrics, which are as follows.

The absolute error is computed by the absolute difference of the predicted result (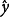) and the actual result (y) for each example in the data set. Mean absolute error (MAE) is the average of absolute errors computed over the data set. Squared error is the square of the difference of the predicted result and actual result for each example in the data set. Mean squared error (MSE) is the average of squared errors computed over the data set. Root mean squared error (RMSE) is the square root of MSE. The mean absolute percentage error (MAPE) is a measure of the prediction accuracy of a forecasting method in statistics, for example, in trend estimation, it is also used as a loss function for regression problems in machine learning. MAPE is preferred for its interpretability as an error metric for forecasts. Equations 9, 10, 11, and 12 show equations for MAE, MSE, RMSE, and MAPE, respectively.(9)
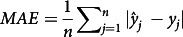

(10)
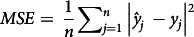

(11)
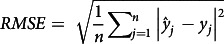

(12)
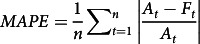
where *A_t_* is the actual value, *F_t_* is the forecast value, and 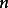 is the total number of observations. The absolute value in this calculation is summed for every forecasted point in time and divided by the number of fitted points, *n*. Multiplying by 100% makes it a percentage error.^16^

## Results

This section is divided into 3 parts. The first section presents the predictions during the phase of lockdown. The second section presents the prediction of total cases after the relaxation of the lockdown. The last section presents the prediction of total deaths. The results in the following sections are based on a confidence interval of 80%.

### Total Cases of COVID-19 during the Lockdown: Forecast Using the Prophet Model

Since March 24, the movements across the states and various activities were restricted when cases were observed to rise. This section analyzes the condition of lockdown until the relaxation of the lockdown and predicts the total cases of COVID-19. The data points that were considered for this section were from March 24 to May 1. This period was considered because this phase observed the most restricted form of lockdown. Hence, for the study of during the lockdown, this period seems the most viable. Furthermore, we have obtained predictions up to May 31. This section is conducted to observe the change in predictions during the corresponding cycle of lockdown and to compare it with the situation after the relaxation of the lockdown. The growth rate would be seen as the most influencing factor to study the comparison.

**Figure 2.**
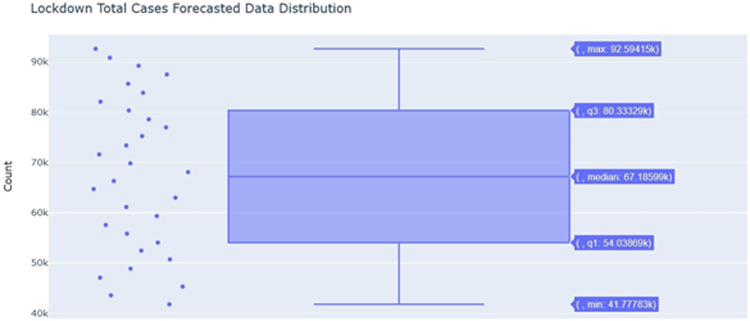
Total cases (lockdown) forecasted in data distribution.

The distribution of the underlying predicted data is displayed of the total cases during the lockdown period, as shown in Figure 2. The scatter points beside it show the data points corresponding to the box plot.

The maximum number (represented as *max* in Figure 2) corresponds to the prediction made on May 31, whereas the minimum number (represented as *min* in Figure 2) corresponds to the prediction made on May 2. Quantiles are defined as split points dividing the range of a distribution into continuous intervals with equal probabilities. In Figure 2, *q1* represents the 25th quantile, median is the value at the 50th quantile, and *q3* represents the 75th quantile.

On May 31, the prediction observed is 92 594 total cases. The prediction rate observed from the prediction is 0.8%. By convention, the period is set to be 15 days, which is half of the horizon period.

The MAPE on average is reported to be 13%, providing an inference of 87% accuracy on the prediction, as stated in Table 2. The other error metrics are higher because a limited amount of data is available for COVID-19. Also, there is no seasonality being observed in the data.

**Table 2.** Performance metrics over the horizon during lockdown – total cases of COVID-19

Error Metric	MSE	RMSE	MAE	MAPE
Mean Value	2200061.29	1095.985	897.3	0.138101

### Cases of COVID-19 after the Lockdown: Forecast Using the Prophet Model

This section considers the dates from March 24 to May 24 and studies the whole of the lockdown period. This set of dates is taken because it is important to study the change in the growth rate and the change in statistics according to the growth rate. The historical data train the model by giving it the idea of upward linearity. Since the cases are rising continuously, the predicted curve tends to show an upward projected path. The holidays’ hyperparameter allows the model to understand those certain dates when the trend is subjected to a downshift due to holidays and therefore the projected value is predicted accordingly. Since the lockdown started on March 24, 2020, and is said to continue until May 31, 2020, with easing the norms of lockdown in multiple phases, the model is initiated with these dates as the lockdown. The first relaxation in the lockdown was observed when trains were allowed for the migrants to travel to their hometowns across the nation on May 1. Since it involves people to be at home, therefore, considering the lockdown as a holiday is justified.

**Figure 3.**
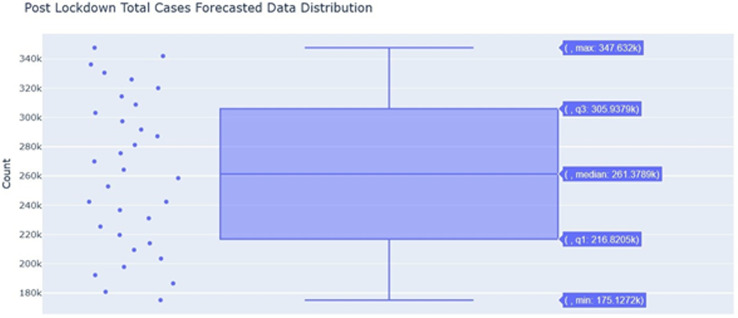
Total cases (post-lockdown) forecasted in data distribution.

As shown in Figure 3, the distribution of the underlying predicted data is displayed for the total cases post-lockdown period. The scatter points beside it show the data points corresponding to the box plot. The maximum number corresponds to the prediction made on June 25, whereas the minimum number corresponds to the prediction made on May 25.

As noticed in the earlier section, the predicted growth rate on May 31 was 0.8%, whereas, in this section on June 25, the growth rate is reported to be 1.6%. This result shows the evidence that relaxation of lockdown is going to make situations unstable. There were certain relaxations made until 24 May, which can be depicted by the number of cases predicted on May 31 being above 200 000, whereas in the earlier section this prediction was 92 594.

The period is set to be 15 days since by convention, it is to be considered half of the horizon. The horizon is 30 days. In Table 3, it has been observed that the model has penalized those dates when the lockdown was imposed, which provides a more accurate prediction. From June 1, 2020, looking at the “lockdown” column, the model is neutral about those dates. Those dates have had relatively higher cases as compared to lockdown dates. Further providing an inference moving to a free lockdown country would result in more cases.

**Table 3.** Forecast of total cases of COVID-19 for 30 days

Dates	Trend	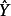 (Predicted Result)	Lockdown
05-25-20	176 574.25	175 127.2	−218.74
05-26-20	182 207.17	180 804.2	−218.74
05-27-20	187 840.09	186 533.9	−218.74
05-28-20	193 473.00	192 212.1	−218.74
05-29-20	199 105.92	197 813.6	−218.74
⁞	⁞	⁞	⁞
06-22-20	334 295.94	330 547.1	0.00
06-23-20	339 928.86	336 223.9	0.00
06-24-20	345 561.77	341 953.8	0.00
06-25-20	351 194.69	347 632.1	0.00

Table 4 depicts the error that was seen on average after the prediction was generated. These errors are higher because there is a less amount of data available about the virus outbreak and its seasonality cannot be predicted upon. Therefore, the model fails in understanding how this trend is affected by seasons. Still, on average, the model is approximately 60% accurate on the predictions and understanding the data. The error seems to be less around 39% as we discuss the 4 days on the horizon with an accuracy 61%.

**Table 4.** Performance metrics over the horizon after lockdown – total cases of COVID-19

Error Metric	MSE	RMSE	MAE	MAPE
Mean Value	5 823 241	1990.913	1324.219	0.40243

### Death Cases of COVID-19 – Forecast Using the Prophet Model

The historical data train the model by giving it the idea of upward linearity. Since the cases are rising continuously, the predicted curve tends to show an upward projected path. The penalty was imposed by the model on the dates of the lockdown due to the holiday parameter. The period considered for computing the prediction of total deaths is made on the complete lockdown, that is, from March 24 to May 24. This period observes relaxation and restrictions on the lockdown period.

**Figure 4.**
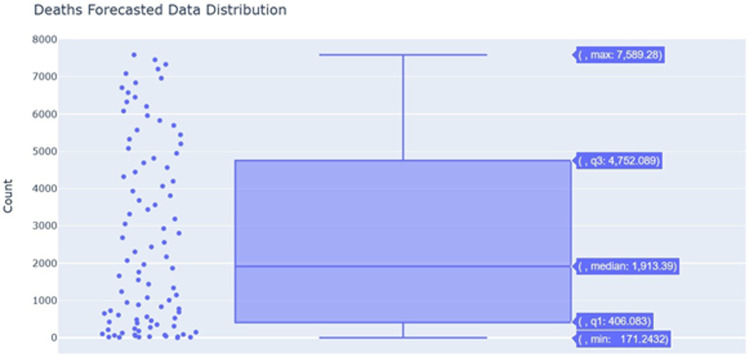
Deaths forecasted in data distribution.

About Figure 4, the distribution of the underlying predicted data is displayed for the total deaths computed over the complete lockdown period. The scatter points beside it show the data points corresponding to the box plot. The maximum number corresponds to the prediction made on June 24, whereas the minimum number corresponds to the prediction made on May 25. The points in between *q1* and *min* show that the model forecasted few numbers of the deaths initially, but they grew apart with the phase of time.

There is not much data about the new coronavirus to consider the seasonality about this virus. Since it has been 4 months from the outbreak, thus there is no need to provide seasonality into the model. The period is set to be 15 days since by convention, it is to be considered half of the horizon. The horizon is 30 days. The model provides 40.51% accuracy for the prediction of death on average. Initially, the error is found to be 28% and the accuracy is 72%. As the day into the forecast increases, the error increases to 75%, making the accuracy fall to 25%.

## Discussion

The new coronavirus has proved to be a global pandemic looking at the number of fatalities and infection it has caused. This research is mainly focused on understanding that, from any such pandemic arising in the future, we can then learn from the safety measures (like lockdown) and how those measures were helpful in dealing with the pandemic in a country like India. The efficiency and results of the future lockdowns can be optimized if we develop a better understanding from the current lockdown in the country. The results in this research have indicated that, during the phase of the lockdown, while having strict measures in place, there were fewer cases predicted and the situation was stable and controlled. While studying and predicting the post-lockdown period, the circumstances are predicted to be uncertain, and prediction shows that the total cases will be uncontrollable in this period because norms have been relaxed. The predicted growth rate of 1.6% as compared to 0.8% illustrates that virus transmission dynamics increases with the relaxation in the course of the lockdown. Therefore, we can observe the high contingency of this virus. The transmission dynamics of high contingency viruses are inversely proportional to the relaxation of the lockdown, as of the time of writing there is no current vaccine or actual treatment available for curing this disease. Other countries that have a comparatively less average population density, as compared to the density in India, can learn from lockdown norms of India on how lockdown helped stabilize the crisis. Even with a better health management system, the severity of total cases in other countries is more as compared to the number of cases in India. Another important point is that, in the initial days of the lockdown, even awareness was not there among the people. The people in the lower strata of the society were not aware of the health implications of this virus and condition of health support in India. These conditions added a more significant challenge in controlling the nationwide spread of the disease. Hence, studying the lockdown aspect further becomes a more necessary factor to examine.

The results in the previous studies have highlighted that the predictions regarding the positive cases are proposed to increase in the coming months. However, based on the findings in this paper, a more plausible explanation can be that relaxation of lockdown, strict norms of lockdown, and self-quarantine are the relevant factors to the control of any high contingency disease. The prediction of total cases and deaths for any disease should depend on these factors because they add more relevance to the predictions, and thus measures could be taken accordingly.

The Prophet model can be fitted on a small amount of data, but the understanding of seasonality is difficult for the model since the data available to the model are of 2 months as mentioned. The sector where the Prophet model shines in this situation is where the analyst can also add change points throughout the period in accordance with the forecast. Change points allow manually adding the points where the model can expect abrupt change in the historical data trend. Another factor that influences the prediction of the model is the holidays component. The lockdown acts as the holiday component, in this case. This model adopts an adaptive seasonality method by default. The accuracy came out to be 87% when the training data were of 1 month because the holidays parameter improved the results. On the other hand, the analysis on the complete phase of the lockdown and predictions of the unlock phase shows that absence of the holiday (lockdown) and change points parameter leads to uncertainty in the prediction as accuracy comes out to be 60%.

With information from other countries, this virus has a mortality rate of 3% where deaths are strongly dependent on the new positive cases. Therefore, the mortality rate in India as well depends strongly on the new cases arising every single day. If we are able to analyze efficiently the historical data based on the total cases and to take measures effectively, then a check can also be kept on the deaths due to this virus.

## Conclusion

The forecasting made by the Prophet model was used to study 2 different situations in this research. During the controlled phase of the lockdown when rules were not relaxed and growth was in control, the model attained an accuracy of 87%, giving confidence that the situation can be normalized after the phase of the lockdown; however, toward the end of the lockdown, the accuracy obtained was 61%, showcasing uncertainty in the unlock phase. Looking at the conclusion from the above 2 scenarios, a lockdown in the future can be optimized accordingly, while observing the rules and regulations that were initially imposed strictly and then later relaxed by the government to control the spread of the pandemic. The number of deaths is very much dependent on the total cases. Post-lockdown period, there is certainly an unprecedented rise in the number of cases. Uncertainty of the number of deaths during the post-lockdown period is predicted because the paradigm is 60% accurate with the forecasts.

It is suggested that when there are seasonal data available, maybe over a period of 1 year or so, this analysis could be conducted again for a better understanding of the transmission dynamics of the virus with optimized measures so that the results can be used to deal with similar pandemic situations in the future.
